# Host Cell Egress and Invasion Induce Marked Relocations of Glycolytic Enzymes in *Toxoplasma gondii* Tachyzoites

**DOI:** 10.1371/journal.ppat.1000188

**Published:** 2008-10-24

**Authors:** Sebastien Pomel, Flora C. Y. Luk, Con J. M. Beckers

**Affiliations:** Department of Cell & Developmental Biology, University of North Carolina, Chapel Hill, North Carolina, United States of America; University of Geneva, Switzerland

## Abstract

Apicomplexan parasites are dependent on an F-actin and myosin-based motility system for their invasion into and escape from animal host cells, as well as for their general motility. In *Toxoplasma gondii* and *Plasmodium* species, the actin filaments and myosin motor required for this process are located in a narrow space between the parasite plasma membrane and the underlying inner membrane complex, a set of flattened cisternae that covers most the cytoplasmic face of the plasma membrane. Here we show that the energy required for *Toxoplasma* motility is derived mostly, if not entirely, from glycolysis and lactic acid production. We also demonstrate that the glycolytic enzymes of *Toxoplasma* tachyzoites undergo a striking relocation from the parasites' cytoplasm to their pellicles upon *Toxoplasma* egress from host cells. Specifically, it appears that the glycolytic enzymes are translocated to the cytoplasmic face of the inner membrane complex as well as to the space between the plasma membrane and inner membrane complex. The glycolytic enzymes remain pellicle-associated during extended incubations of parasites in the extracellular milieu and do not revert to a cytoplasmic location until well after parasites have completed invasion of new host cells. Translocation of glycolytic enzymes to and from the *Toxoplasma* pellicle appears to occur in response to changes in extracellular [K^+^] experienced during egress and invasion, a signal that requires changes of [Ca^2+^]_c_ in the parasite during egress. Enzyme translocation is, however, not dependent on either F-actin or intact microtubules. Our observations indicate that *Toxoplasma gondii* is capable of relocating its main source of energy between its cytoplasm and pellicle in response to exit from or entry into host cells. We propose that this ability allows *Toxoplasma* to optimize ATP delivery to those cellular processes that are most critical for survival outside host cells and those required for growth and replication of intracellular parasites.

## Author Summary

Protozoan parasites of humans are important causes of disease throughout the world. Amongst these, the apicomplexan parasites are an especially important group of pathogens, as they include *Plasmodium* species, the causative agents of malaria, and *Toxoplasma gondii*, an important cause of disease in people with AIDS. These parasites divide their stay in the infected human into two discrete stages, the motile extracellular stage that is responsible for parasite spread throughout its host, and the intracellular stage that is devoted entirely to replication. Here we demonstrate that *Toxoplasma* relocates its main source of energy when it moves between the intracellular and extracellular environment. It appears that this allows the parasite to optimize energy delivery to those processes that are critical for extracellular survival and invasion into new host cells and those required for subsequent intracellular growth and replication.

## Introduction

*Toxoplasma gondii* is an obligate intracellular protozoan parasite of humans and other warm-blooded animals, and is closely related to *Plasmodium sp.*, the causative agent of malaria. Although the majority of *Toxoplasma* infections in humans are asymptomatic, severe disease and death can occur in developing fetuses and in immunocompromised individuals. Infection of and spread between host cells by *Toxoplasma* and its close relatives *Plasmodium*, *Cryptosporidium*, and *Eimeria* is critically dependent on actin-myosin based motility systems in the parasite.

The actin filaments required for host cell invasion and for general parasite motility are believed to be associated with the cytoplasmic tails of adhesins in the parasite plasma membrane. Specifically, the microneme proteins thrombospondin-related anonymous protein (TRAP) of *Plasmodium* sp. and MIC2 of *Toxoplasma* are believed to interact with F-actin through the glycolytic enzyme aldolase-1 [1],[2]. Myosin-A, a type XIV myosin, is also critical for gliding motility [3]. This protein is found in a complex with an atypical myosin light chain [4] and two accessory proteins, GAP45 and the integral membrane glycoprotein GAP50 [5], of which the latter is responsible for anchoring the motor complex in the parasite's inner membrane complex (IMC). This organelle consists of flattened membrane cisternae that are closely apposed to the parasite plasma membrane and extends from the anterior end of the parasite to the posterior end. The only obvious connection between the main body of the parasite cytoplasm and the space between its plasma membrane and IMC is found at the extreme anterior end of the parasite. At this location, the IMC is clearly interrupted, creating a gap through which the conoid and rhoptries protrude [6]. It is also believed that it is through this gap that the parasites' micronemes access and fuse with the plasma membrane.

Both actin polymerization and myosin action are ATP-dependent processes. Most eukaryote cells are capable of generating ATP through multiple mechanisms: glycolysis and the oxidative phosphorylation of acetyl-CoA derived from pyruvate, β-oxidation of fatty acid, or from certain amino acids. It is evident that apicomplexan parasites possess a complete glycolytic pathway [7] as well as all enzymes for the TCA cycle and mitochondrial electron transport chain. It was therefore very surprising that the only obvious homolog of pyruvate dehydrogenase in *Plasmodium* and *Toxoplasma* is targeted to the apicoplast, a plastid-like organelle, along with several glycolytic enzymes [7]–[9]. In other eukaryotes, pyruvate dehydrogenase is targeted to mitochondria where it is involved in the conversion of glycolysis-derived pyruvate into acetyl-CoA and thus occupies a critical link between glycolysis and oxidative phosphorylation. The apparent absence of pyruvate dehydrogenase from apicomplexan mitochondria therefore suggests that glycolysis and oxidative phosphorylation are not coupled in *Toxoplasma* and *Plasmodium sp.* It remains possible, however, that these organisms have evolved a structurally distinct pyruvate dehydrogenase. In other cells, acetyl-CoA can also be produced through the β-oxidation of fatty acids or the catabolism of amino acids. This process is also unlikely to occur in apicomplexan parasites as, at least in the case of *Plasmodium sp*., the genome does not appear to encode the requisite enzymes for these processes [9].

In this study, we investigated the likely sources of the ATP that is critical for gliding motility of *Toxoplasma* tachyzoites. Our data indicate that glycolysis is the major source of energy for parasite motility and that oxidative phosphorylation, if it occurs at all, makes only a minor contribution. We also demonstrate that the glycolytic enzymes of *Toxoplasma* are distributed smoothly over the cytoplasm of intracellular parasites. In a surprising finding, we found that the glycolytic enzymes undergo a translocation from the parasite cytoplasm to the pellicle upon egress from host cells and remain pellicle-associated throughout the extracellular phase of *Toxoplasma* and during invasion of host cells. Only after host cell invasion is complete do the enzymes revert to a smooth cytoplasmic distribution. This translocation of the glycolytic enzymes between the parasite cytoplasm and the pellicle appears to be triggered by the changes in potassium concentrations experienced by the parasites during egress from and invasion into host cells. These findings suggest that *Toxoplasma gondii* tachyzoites are able to relocate their major source of ATP production to the site where energy is required at each stage during their life cycle.

## Results

### Glycolysis is a major source of energy for *Toxoplasma* motility

**Figure 1:**
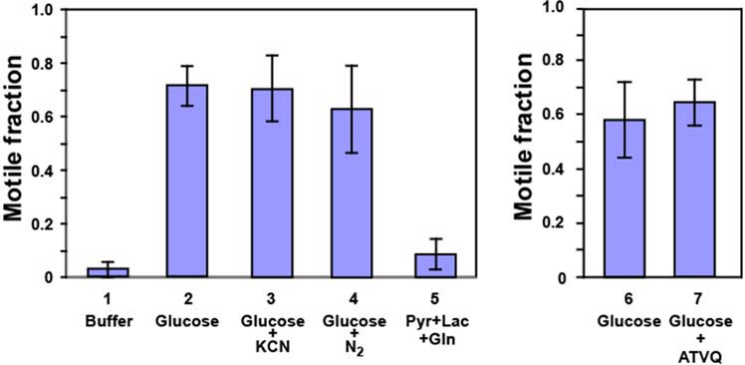
Glycolysis is the primary source of energy for *Toxoplasma* motility. *Toxoplasma* tachyzoites were isolated from an infected monolayer 30–32 hours after infection as described in Materials and Methods. Parasites were collected by centrifugation and resuspended in EC buffer containing no carbon source or in the presence of glucose, glucose and KCN, or a mixture of pyruvate, lactate and glutamine. The effect of 10 nM atovaquone on *Toxoplasma* motility was analyzed in separate experiments. Incubations were performed in air or under a nitrogen atmosphere for 15 minutes at 37°C. Motility experiments were performed in quadruplicate and analyzed as described in Materials and Methods. The average fraction of motile parasites (±S.D.) under the different incubation conditions is shown.

*Toxoplasma* invasion into and egress from its animal host cells are dependent on an actin-myosin-based motility system and thus require ATP for myosin action as well as actin filament formation. In most cells, the ATP necessary for these processes can be generated by glycolysis and by oxidative phosphorylation. The *Toxoplasma* genome encodes all the enzymes required for ATP production by glycolysis. It is not clear, however, whether apicomplexan parasites can produce ATP by oxidative phosphorylation given that pyruvate dehydrogenase, a key enzyme in coupling glycolysis and oxidative phosphorylation, appears to be absent from the parasite's mitochondria [7]–[11] as are some subunits of the ATP synthase [12],[13]. In order to better understand the roles of both metabolic pathways in supplying energy for parasite movement, we performed parasite motility assays under conditions where the parasites either had to rely on glycolysis for most of their energy production or on oxidative phosphorylation. Our observations are summarized in Figure 1. When assays were performed in the presence of glucose, a carbon source for both glycolysis and oxidative phosphorylation, we observed robust movement in a majority of the parasites (71.6±7.4%). Omission of glucose resulted in a drastic decrease in the fraction of mobile parasites (2.7±2.9%). When experiments were performed in the presence of glucose but under a nitrogen atmosphere or in the presence of KCN, conditions where any oxidative phosphorylation should be inhibited, *Toxoplasma* motility was not significantly affected with 70.5±12.4% and 63.1±16.1% of parasites moving, respectively. Although these observations indicate already that glycolysis is the major source of energy for parasite movement, they do not exclude the possibility that oxidative phosphorylation does make some contribution. To test this possibility, *Toxoplasma* motility experiments were performed in the absence of glucose but in the presence of potential substrates for the parasites' TCA cycle: pyruvate, lactate, or glutamine, both individually and in combination. As can be seen in Figure 1, only a small fraction of the parasites demonstrated motility (8.4±5.8%) and, as we observed in the absence of any substrate, this was limited to trails that were barely longer than the 2 µm cut-off limit (see Materials and Methods).

Additional evidence against a role for oxidative phosphorylation in generating energy for *Toxoplasma* motility was derived from motility experiments performed in the presence or absence of atovaquone, a potent antiparasitic agent that acts as an inhibitor of the cytochrome bc_1_ complex in apicomplexan parasites [14]–[16]. As can be seen in Figure 1, atovaquone does not have an obvious effect on *Toxoplasma* motility at a concentration of 0.5 µM in our typical experimental setup. Prolonged treatment of intracellular *Toxoplasma* with the same concentration atovaquone resulted in parasite death as had been previously observed [14]–[16] (data not shown).

Together, these observations suggest that glycolysis is the major source of energy for *Toxoplasma* motility and that oxidative phosphorylation, if it occurs at all, makes at best a very small contribution.

### The *Toxoplasma* glucose transporter GT1 is found throughout the plasma membrane

**Figure 2:**
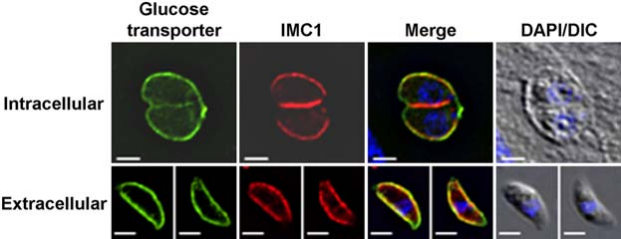
The glucose transporter GT1 is found in the plasma membrane of *Toxoplasma gondii* tachyzoites. The subcellular location of the myc-tagged glucose transporter GT1 in intracellular and extracellular *Toxoplasma* tachyzoites was determined by immunofluorescence microscopy using antibodies to the myc-epitope (green) and to the membrane skeleton protein IMC1 (red). Overlays of DIC and DAPI fluorescence images are shown on the right. Bars = 2 µm.

The plasma membrane of apicomplexan parasites is found in close association with large flattened cisternae, referred to as the inner membrane complex or IMC. The IMC extends beneath the entire plasma membrane of the parasites, except at the extreme anterior end where the plasma membrane is in direct contact with the cytoplasm (Mann and Beckers, 2001). The space between the plasma membrane and IMC is only 18.4±0.6 nm (Figure S1) and no trans-IMC transport mechanisms have been identified to date. It is therefore not clear whether nutrients like glucose enter parasites only at the anterior end, where there is direct contact between the plasma membrane and the cytoplasm, or if import occurs all over the parasite plasma membrane. To test which of these scenarios is used for glucose acquisition, we expressed a myc-tagged version of the *Toxoplasma* hexose transporter TgGT1 in the parasite. TgGT1 is a high affinity facilitative hexose transporter [17] and appears to be the only hexose transporter encoded by the *Toxoplasma* genome. As can be seen in Figure 2, TgGT1 is found exclusively in the parasite plasma membrane and is distributed evenly over the parasite surface. This result suggests that glucose transport into the parasite is not confined to a particular part of its plasma membrane.

### The subcellular location of aldolase-1 is different in intra and extracellular *Toxoplasma*

The actin-myosin XIV based motile apparatus of apicomplexan parasites is found in the narrow space between the parasite plasma membrane and IMC which extends from the anterior to the posterior part of the organisms [1],[5]. Given that parasite motility appears to be powered primarily by glycolysis and that the glucose transporter TgGT1 is found along the entire surface of *Toxoplasma* tachyzoites (Figure 2), we wondered whether part or all of the *Toxoplasma* glycolytic apparatus was also present in close proximity to the parasite pellicle and therefore its motile apparatus. Jewett and Sibley had, in fact, shown that a fraction of the glycolytic enzyme aldolase is found in this space [1]. Specifically, aldolase was reported to form a bridge between F-actin and the cytoplasmic tail of the adhesin MIC2. Given the critical role of aldolase in glycolysis, we sought to repeat these observations. As the observations of Jewett and Sibley were based on cross-reactivity of commercial anti-rabbit aldolase antisera with *Toxoplasma* aldolase, we initially employed similar antisera. The *Toxoplasma* genome contains two potential aldolase-encoding sequences, but only one of these, aldolase-1, is expressed in *Toxoplasma* tachyzoites and the second appears to represent a pseudogene ([7], data not shown). As the commercial anti-rabbit aldolase antisera do not cross-react with *Toxoplasma* aldolase-1 in our hands (Figure S2A), we generated an antiserum that is specific to *Toxoplasma* aldolase-1 and that does not cross-react with mammalian aldolases (Figure S2B). As an alternative strategy, we also generated a *Toxoplasma* line that stably expresses myc-tagged *Toxoplasma* aldolase-1.

As can be seen in Figure S2B, the anti-*Toxoplasma* aldolase-1 antiserum reacts with a single protein of the expected MW_app_ in immunoblots of parasite lysates. In parasites expressing myc-tagged aldolase-1, the antiserum detects the same protein as in non-transfected parasites, as well as a slightly larger protein species that represents the myc-tagged enzyme. A monoclonal anti-myc antibody only reacts with the larger protein species, which is only present in parasites expressing myc-aldolase-1. Based on the blot with anti-aldolase-1, it appears that the myc-aldolase-1 is expressed at a level that is approximately three times higher than endogenous aldolase-1.

**Figure 3:**
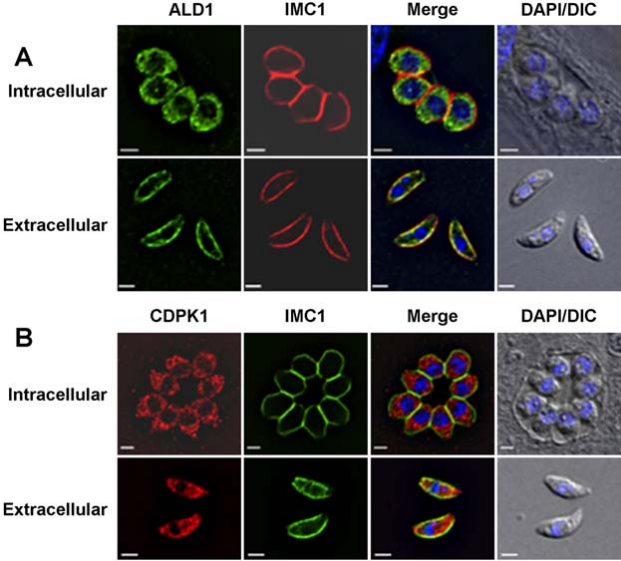
Endogenous aldolase-1 is cytoplasmic in intracellular *Toxoplasma* and associated with the pellicle of extracellular tachyzoites. The subcellular locations of (A) endogenous aldolase-1 (ALD1) and (B) the cytoplasmic protein kinase CDPK1 were determined in intracellular and extracellular *Toxoplasma* fixed in −20°C methanol and compared to the membrane skeleton protein IMC1. Note that whereas aldolase-1 localizes differently in intracellular and extracellular parasites, the distribution of CDPK1 does not differ substantially. The nuclei were visualized in both cases with DAPI and are shown in overlays with DIC images. Bars = 2 µm.

**Figure 4:**
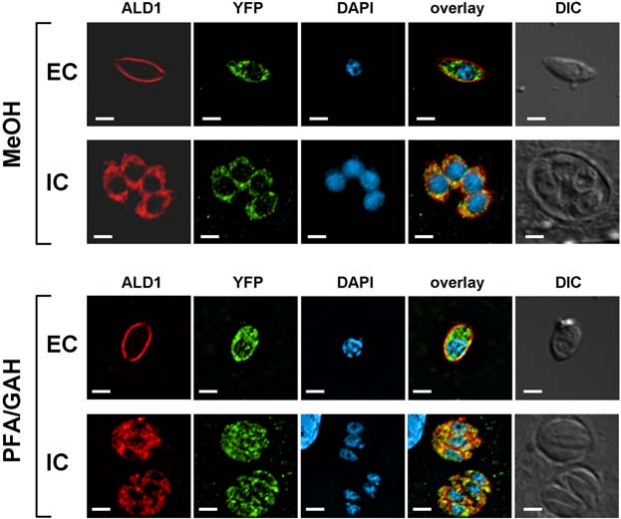
Different fixation conditions do not affect aldolase-1 distribution. To ensure that the observed redistribution of aldolase-1 is not a fixation artifact we also compared the distribution of aldolase-1 and cytoplasmic YFP in extracellular (EC) and intracellular (IC) *Toxoplasma* fixed in methanol (MeOH) or 3% paraformaldehyde/0.01% glutaraldehyde (PFA/GAH). Merged images of the various proteins and the parasite nuclei are shown as well as DIC images. Bars = 2 µm.

Jewett and Sibley found that the anti-rabbit aldolase antiserum reacted with material found in the anterior part of the parasite and that this signal overlapped substantially with that of the microneme protein MIC2 [1]. When we used our affinity-purified antibodies to *Toxoplasma* aldolase-1 to localize this enzyme in intracellular parasites, we found it to be distributed throughout the parasites' cytoplasm (Figure 3A and Figure 4), irrespective of the fixation conditions used. In extracellular parasites, on the other hand, the protein localized to the parasite periphery (Figure 3A, C) where it overlapped with IMC1, a membrane skeleton protein of the *Toxoplasma* IMC [18]. The peripheral location of the *Toxoplasma* aldolase-1 in extracellular parasites is specific; other cytoplasmic proteins, such as the calcium-dependent protein kinase CDPK1 [19] (Figure 3B) and YFP (Figure 4), retain their normal cytoplasmic distribution in both intracellular and extracellular parasites. The observed redistribution of aldolase-1 is also unlikely to be a fixation artifact. We compared the distribution of both aldolase-1 and YFP in intra and extracellular parasites fixed with −20°C methanol or paraformaldehyde and glutaraldehyde. As can be seen in Figure 4, aldolase-1 is found at the pellicle of extracellular *Toxoplasma* and in the cytoplasm of intracellular parasites, irrespective of the fixation condition used. As expected, the cytoplasmic distribution of YFP was also not affected by the fixation method used.

**Figure 5:**
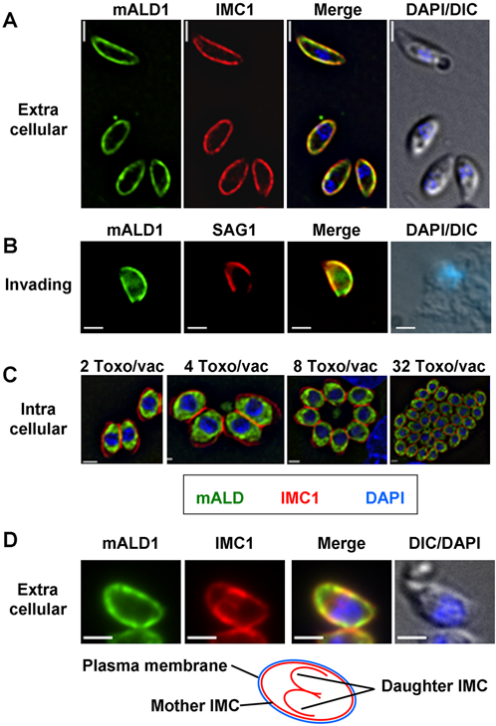
Distribution of aldolase-1 in extracellular, invading, and intracellular *Toxoplasma*. (A–D) Immunofluorescence microscopy was used to determine the distribution of myc-tagged aldolase-1 (mALD1, green) in (A, D) extracellular parasites, (B) invading parasites, and (C) intracellular parasites immediately after invasion or after 1, 2, 3, and 5 rounds of replication. Panel D demonstrates the distribution of aldolase-1 in extracellular parasites caught in the process of endodyogeny. Note the selective association of the enzyme with only the IMC of the mother parasite and not that of the immature daughter cells. All panels show parasites and parasite-infected cells fixed in −20°C methanol. In panels A and D, parasites were counterstained with antibodies to the membrane skeleton protein IMC1 (red). In panel B, parasites expressing myc-aldolase-1 were allowed to interact with HFF cells for 2 minutes at 37°C, followed by the decoration of extracellular parts of the parasites with anti-SAG1 antiserum (red), which was in turn followed by cell fixation and permeabilization and staining with anti-myc monoclonal antibody (green). Parasite nuclei were visualized using DAPI (blue). Bars = 2 µm.

To further assure ourselves that we were observing a relocation of aldolase-1, we repeated the experiments in Figure 3A with parasites expressing myc-tagged aldolase-1. As can be seen in Figure 5A and C, myc-aldolase-1 displays a similar change in distribution between intracellular and extracellular parasites. It is interesting to note that we did not observe an overlap between the aldolase-1 or myc-aldolase-1 signal with that of the microneme protein MIC2 (Figure S3) which stands in contrast to the previous observations [1].

### Subcellular distribution of *Toxoplasma* aldolase-1 during the tachyzoite life cycle

Given the striking difference in aldolase-1 distribution we observed between intracellular and extracellular *Toxoplasma* tachyzoites, we sought to determine if the redistribution of this enzyme occurred at a specific stage in parasite development or a particular point in parasite-host cell interaction.

**Figure 6:**
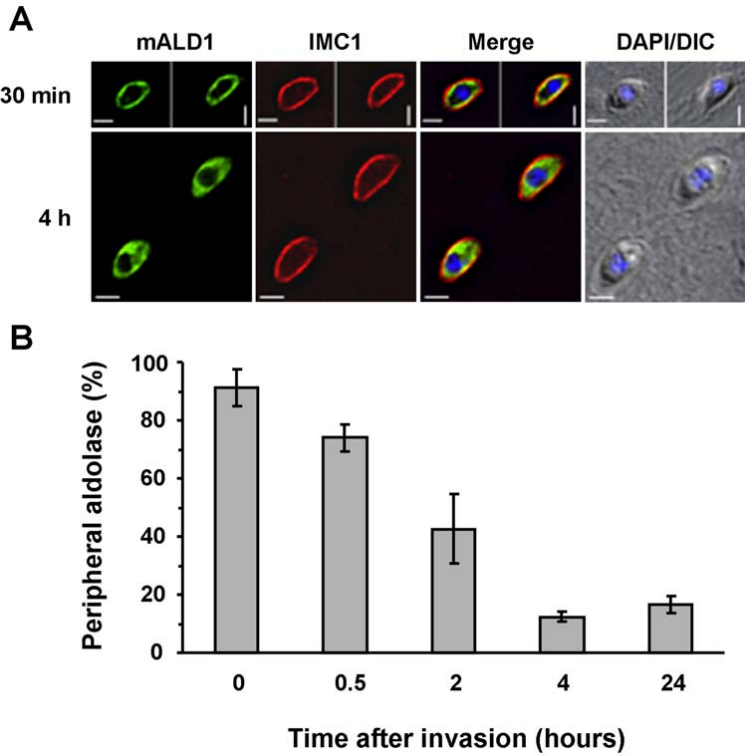
Quantitative analysis of aldolase-1 distribution in *Toxoplasma*. Freshly egressed parasites expressing myc-aldolase 1 were allowed to attach to HFF monolayers for 5 minutes at 37°C in complete medium. Non-attached parasites were washed off and the incubation continued in complete medium at 37°C for the indicated times. (A) The distribution of aldolase-1 30 minutes and 4 hours after invasion of host cells. Overlays are shown of myc-aldolase-1 (mALD1, green), the IMC protein IMC1 (red). Overlay pictures of DIC and DAPI blue) are shown on the right. Parasite-infected cells were fixed in −20°C methanol. Images of the individual channels are shown in Figure S4. Bars = 2 µm. (B) A quantitative analysis of the results is shown. Data represent averages of 32 fields counted over 4 samples. Error bars indicate the standard deviation.

As *Toxoplasma* aldolase-1 reverts to a smooth cytoplasmic distribution at some point after host cell invasion we first tested whether this occurred during or shortly after host cell invasion, or if it occurred at a later stage. We therefore allowed freshly isolated extracellular parasites to interact with new host cells for 2 minutes and monitored the aldolase-1 distribution immediately after this incubation and after various lengths of incubation at 37°C. Extracellular and intracellular parasites could be distinguished by the inability of the latter to be recognized by an antibody to the surface antigen SAG1 in intact host cells. After short incubations, numerous parasites were captured in the process of invading host cells as judged by the fact that only a part of their surface reacted with the SAG1 antiserum (Figure 5B). In these parasites, the aldolase-1 remains located at the parasite periphery. The aldolase-1 distribution was also not affected in parasites that had just completed host cell invasion (Figure 6A). The peripheral location of the aldolase-1 was maintained in a large fraction (74±5%) of parasites at 30 minutes after invasion but decreased to less than 10% after 4 hours (Figure 6B) and was no longer detectable after parasites had undergone one round of replication (Figure 5C; Figure S4). The cytoplasmic distribution of aldolase-1 was maintained as long as the parasites remained inside host cells, irrespective of the number of parasites per vacuole or the stage of parasite replication (Figure 5C; Figure S4).

The pellicle of *Toxoplasma* consists of three membranes, the plasma membrane and the membranes of the IMC. During parasite replication, two immature daughter parasites are formed in the cytoplasm of the mother cell. These daughters possess all structural elements of the mature parasite except for the plasma membrane, which they only acquire from the mother parasite at the final stage of replication. Typical preparations of extracellular *Toxoplasma* always contain a few parasites that are released from host cells at various stages of daughter cell formation. When the distribution of aldolase-1 was analyzed in such parasites, it was evident that the enzyme is only associated with the pellicle of mature parasites and not with the IMC of the immature daughters (Figure 5D). This finding suggests that IMC association of aldolase-1 is not only controlled by the parasite's environment but is also under strict developmental control by limiting pellicle association to mature parasites only.

### Regulation and mechanism of aldolase-1 redistribution during host cell invasion and egress

We considered the possibility that the change in location of aldolase-1 between intracellular and extracellular *Toxoplasma* reflected the proteolytic destruction of preexisting enzyme pool and the synthesis of a novel pool of protein that was targeted to a new location, possibly through differences in post-translational modifications. Pretreatment of parasites with 10 µM cycloheximide, a concentration sufficient to block >98% of de novo protein synthesis in *Toxoplasma* [19], did not affect translocation of aldolase-1 to the pellicle upon egress or its translocation to the cytoplasm after host cell invasion (data not shown), indicating that we are indeed detecting the translocation of preexisting enzyme.

**Figure 7:**
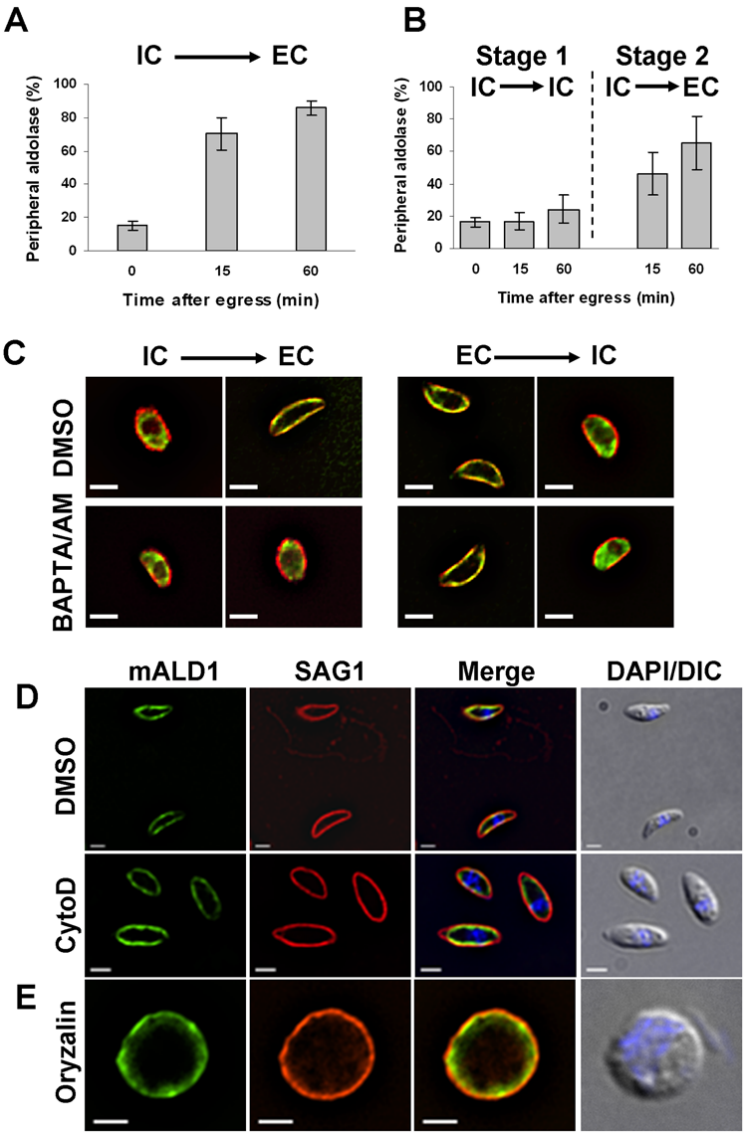
Aldolase-1 relocation during egress requires a decrease in environmental [K^+^] and an increase in [Ca^2+^]_c_ and does not require either F-actin or microtubules. (A) Intracellular parasites expressing myc-aldolase-1 were harvested 24 hours after infection in IC buffer as described in Materials and Methods and subsequently incubated for the indicated times in IC buffer at 37°C. After 60 minutes in IC buffer, parasites were recovered by centrifugation, resuspended in EC buffer, and incubated at 37°C for the indicated time. The fraction of parasites with peripheral aldolase-1 was determined as described above. (B) Intracellular parasites expressing myc-aldolase-1 were harvested 24 hours after infection in EC buffer as described in Materials and Methods and subsequently incubated for the indicated times in EC buffer at 37°C before fixation and processing for immunofluorescence microscopy as in Figure 4. The fraction of parasites with peripheral aldolase-1 was determined as in panel A. (C) Intracellular parasites expressing myc-tagged aldolase-1 were released from host cells in IC buffer and subsequently switched to EC buffer. Extracellular parasites expressing myc-tagged aldolase-1 were resuspended in EC buffer and further switched to IC buffer. Each buffer was supplemented by DMSO (control) or BAPTA-AM (20 µM). Parasites were incubated in each buffer for 30 min at 37°C and processed for immunofluorescence using mouse anti-myc antibody (green) and rabbit anti-IMC1 antiserum (red). Bars = 2 µm. (D) Intracellular parasites expressing myc-tagged aldolase-1 were treated with DMSO or 10 µM cytochalasin D (CytD) for 15 min at 37°C and subsequently released from host cells by passage through a 25G needle. Motility assays were performed in the presence of DMSO or cytochalasin D as described in Materials and Methods. Parasites were labeled using anti-myc (mALD1, green) and anti-SAG1 (red) antibodies. In the top panel, note the presence of a motile (top) and a non-motile (bottom) parasite. No motility was detected after cytochalasin D treatment. Overlay pictures of DIC and DAPI are shown on the right. Bars = 2 µm. (E) Intracellular parasites were treated for 24 hours with 2.5 µM oryzalin and subsequently released from host cells by passage through a 25G needle into EC buffer. After 15 minutes at 37°C, parasites were fixed and processed for immunofluorescence microscopy using anti-myc (mALD1, green) and anti-IMC1 (red) antiserum. An overlay of the DIC and DAPI are shown on the right. All samples were fixed in −20°C methanol. Bar = 2 µm.

It appears that translocation of aldolase-1 from the cytoplasm to the pellicle only occurs at or shortly after *Toxoplasma* egress from its host cells. Egress is not synchronous or predictable under physiological conditions but is readily induced by the physical disruption of the host cells [20]. Although earlier time points could not be obtained for practical reasons, aldolase-1 translocation to the *Toxoplasma* pellicle could be detected as early as 30 seconds after parasite egress and was essentially complete by 60 minutes (Figure 7A).

This finding implies that the parasite can sense whether it is inside or outside of a host cell and can translocate aldolase and presumably the rest of its glycolytic apparatus accordingly. We have previously described that parasite egress from its decaying host cells is triggered by the reduction in the [K^+^] of the surrounding host cytoplasm, which in turn results in an increase in cytoplasmic [Ca^2+^] [20]. As the relocation of *Toxoplasma* aldolase-1 also occurs upon host cell egress, we wondered whether it might also be controlled by changes in environmental [K^+^]. We therefore broke *Toxoplasma*-infected HFF cells in media containing [K^+^] consistent with the environment inside or outside animal host cells and monitored changes in aldolase-1 distribution. As can be seen in Figure 7A, disruption of parasite-infected HFF cells in a medium mimicking the extracellular environment (EC medium) results in translocation of aldolase-1 to the parasite pellicle with half-maximal translocation observed at about 15 minutes. When parasite-infected host cells are disrupted in medium with a high [K^+^] (IC medium) this translocation does not occur and aldolase-1 remains distributed evenly over the parasite cytoplasm (Figure 7B, stage 1). This effect is entirely reversible in that a subsequent exposure to extracellular medium results in rapid translocation of aldolase-1 to the parasite pellicle (Figure 7B, stage 2). These findings indicate that translocation of aldolase-1 from the *Toxoplasma* cytoplasm to its pellicle is controlled by the [K^+^] in the surrounding medium and requires increases in [Ca^2+^].

The transfer of aldolase-1 from the parasite periphery to the cytoplasm that we observed after host invasion also appears to be triggered by a change in extraparasitic [K^+^]. When extracellular *Toxoplasma* are transferred to a medium with [K^+^] similar to the intracellular milieu, aldolase-1 is transferred from the parasite pellicle to its cytoplasm (Figure 7C). As was observed during normal infection of host cells, this occurred with substantially slower kinetics than the forward reaction. Reversal had occurred in only ∼60% of parasites after 1 hour in high [K^+^] medium and was essentially complete after 4 hours. The translocation of aldolase-1 from the *Toxoplasma* pellicle to its cytoplasm in response to high [K^+^] medium was also entirely reversed upon transfer of parasites to EC medium (data not shown).

Activation of *Toxoplasma* motility in response to a reduction of environmental [K^+^] requires an increase in [Ca^2+^]_c_ in the parasite [20]. To determine if an increase in [Ca^2+^]_c_ is also required to trigger translocation of glycolytic enzymes in response to [K^+^] changes, we incubated parasites for 30 minutes in high [K^+^] medium with BAPTA/AM, a chelator of cytoplasmic Ca^2+^, followed by a 30 minute incubation in extracellular medium containing the same compound. Chelation of cytoplasmic Ca^2+^ did not affect the cytoplasmic distribution of *Toxoplasma* aldolase-1 while in high [K^+^] medium (Figure 7C). When the BAPTA/AM-treated parasites were subsequently transferred to EC medium, the aldolase-1 remained evenly distributed throughout the parasite cytoplasm whereas aldolase-1 in mock-treated parasites had translocated to the pellicle (Figure 7C).

When parasites that had been previously incubated in EC medium and uniformly displayed pellicle-associated aldolase-1 were subsequently treated with BAPTA/AM, we did not observe any change in pellicle-association of aldolase-1 compared to untreated parasites (Figure 7C). When these BAPTA/AM-treated parasites were subsequently transferred to IC medium, aldolase-1 reverted to a cytoplasmic distribution that was indistinguishable from untreated parasites (Figure 7C).

Aldolase-1 translocation from the parasite cytoplasm to its pellicle does not require either F-actin or microtubules. When intracellular parasites were pre-treated with cytochalasin-D and subsequently released from their host cells, aldolase-1 relocation to the parasite pellicle was not affected (Figure 7D). Disruption of *Toxoplasma* microtubules with oryzalin had, as described before [21], a profound effect on parasite morphology but also did not affect aldolase-1 translocation to the pellicle after egress from host cells (Figure 7E). As cytochalasin-D and oryzalin-treated parasites are incapable of host cell invasion [22],[23], we could not determine whether F-actin or microtubules played a role in the pellicle-to-cytoplasm translocation after this process.

Pellicle association of aldolase-1 in intact extracellular *Toxoplasma* is very stable, even during incubations of up to 24 hours at 37°C in complete medium. The distribution of the enzyme in the parasite pellicle was also not noticeably affected during this incubation (data not shown). Jewett and Sibley had reported that *Toxoplasma* aldolase appeared to undergo an anterior-to-posterior translocation in moving parasites [1]. We did not observe such a translocation irrespective of whether we monitored the endogenous enzyme with anti-*Toxoplasma* aldolase-1 antiserum or the myc-aldolase-1; the enzyme was found evenly distributed along the pellicle of both motile and non-motile parasites (Figure 7D).

These observations demonstrate that the translocation of aldolase-1 from the *Toxoplasma* cytoplasm to its pellicle is triggered by the decrease in [K^+^] surrounding the parasites in compromised host cells and that this response is mediated by an increase in [Ca^2+^]_c_ in the parasite. Aldolase-1 translocation from the parasite pellicle to its cytoplasm upon host cell invasion is also triggered by the increased [K^+^] in its environment. Unlike what occurs during parasite egress, however, this process does not appear to be mediated by an increase in [Ca^2+^]_c_ in the parasite. Although the molecular mechanism underlying aldolase-1 translocation is not known at this time, it is clear that neither F-actin nor microtubules are essential.

### Localization of other glycolytic enzymes in intra and extracellular *Toxoplasma*

**Figure 8:**
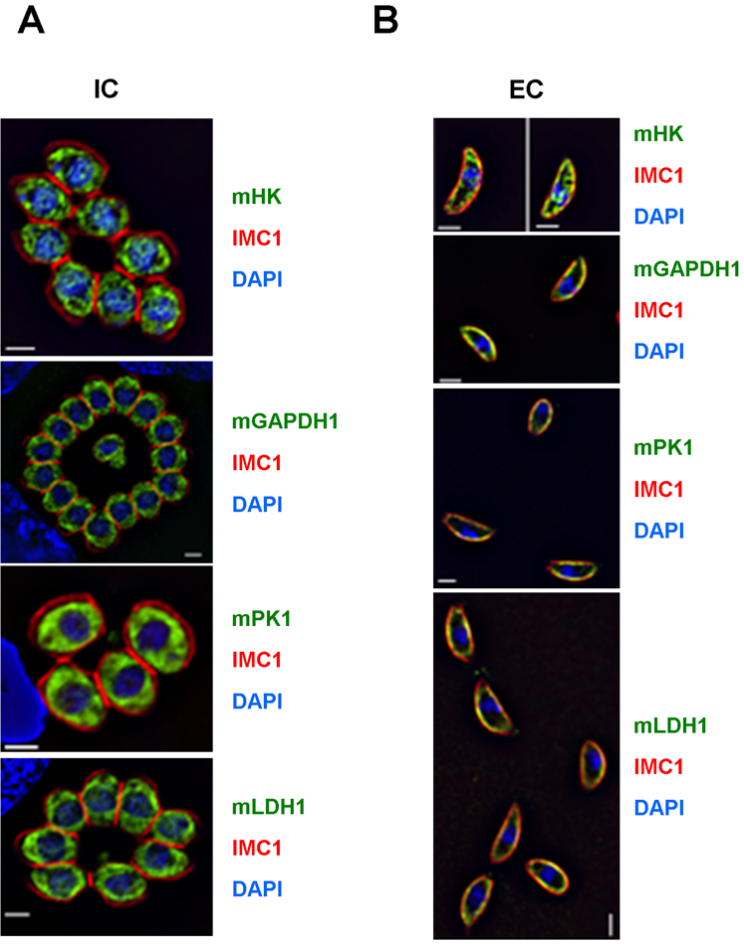
Redistribution of other glycolytic enzymes to the pellicle of extracellular parasites. The subcellular location of myc-tagged hexokinase (mHK), glyceraldehyde-3-phosphate dehydrogenase-1 (mGAPDH1), pyruvate kinase-1 (mPK1), or lactate dehydrogenase-1 (mLDH1) was determined in (A) intracellular and (B) extracellular *Toxoplasma* tachyzoites by immunofluorescence microscopy after fixation in −20°C methanol. The IMC of the parasites was visualized using antibodies to IMC1 (red) and their nuclei using DAPI (blue). The separate images are shown in Figure S5. Bars = 2 µm.

Although we did not detect any redistribution of aldolase-1 in motile *Toxoplasma* tachyzoites and the translocation of aldolase-1 to the parasite periphery was not affected by cytochalasin-D (Figure 7D), these findings do not necessarily conflict with a role of peripheral aldolase-1 in providing a link between the adhesin MIC2 and F-actin as was suggested by Jewett and Sibley [1]. We did, however, want to consider an alternative possibility in which translocation of aldolase-1 from the *Toxoplasma* cytoplasm to its periphery was only one element in the relocation of the entire glycolytic apparatus and hence a major source of ATP. To test this model, we expressed epitope-tagged versions of other enzymes along the glycolytic pathway of *Toxoplasma* tachyzoites and monitored their distribution in intracellular and extracellular parasites. As can be seen in Figure 8 and Figure S5, enzymes at the beginning (hexokinase), in the middle (glyceraldehyde-3-phosphate dehydrogenase-1), and at the end of the glycolytic pathway (pyruvate kinase-1) demonstrated subcellular distribution patterns that are very similar to those of aldolase-1. All three enzymes are found throughout the cytoplasm of intracellular *Toxoplasma* and associated with the pellicles of extracellular parasites.

During glycolysis, a single NAD^+^ is converted to NADH for each conversion of glyceraldehyde-3-phosphate to 1,3-bisphosphoglycerate. As cells contain only a limited amount of NAD^+^ this critical compound needs to be regenerated constantly to ensure continuation of glycolysis. In the absence of oxidative phosphorylation, NAD^+^, is regenerated in most eukaryotes by lactic acid fermentation. In this process, the end product of glycolysis, pyruvate, is converted to lactic acid by lactate dehydrogenase (LDH), converting a single NADH to NAD^+^ in the process. Given that the data in Figure 8B demonstrate that all tested glycolytic enzymes in extracellular *Toxoplasma* are found in close association with the parasite's pellicle, we wondered whether *Toxoplasma* LDH was found in the same location. The *Toxoplasma* genome encodes two isoforms of this enzyme, LDH1 and LDH2, of which the latter is only expressed in bradyzoites [24]. When myc-tagged *Toxoplasma* LDH1 was expressed in tachyzoites we found that, like the glycolytic enzymes, this protein is also found in the cytoplasm of intracellular parasites and in close association with the pellicle in extracellular organisms (Figure 8).

In summary, hexokinase, aldolase-1, glyceraldehyde-3-phosphate dehydrogenase-1, pyruvate kinase, and lactate dehydrogenase-1 translocate from the *Toxoplasma* cytoplasm to its pellicle upon parasite egress, and back to the cytoplasm after host cell invasion. This finding suggests that all enzymes required for ATP production by glycolysis are translocated in a concerted fashion between the *Toxoplasma* cytoplasm and its pellicle depending on whether the parasite is inside or outside of its host cell.

### Pellicle association of glycolytic enzymes in *Toxoplasma*

To characterize this pellicle association in more detail we fractionated *Toxoplasma* homogenates at different salt concentrations and monitored the distribution of aldolase-1 between the soluble and insoluble fractions.

**Figure 9:**
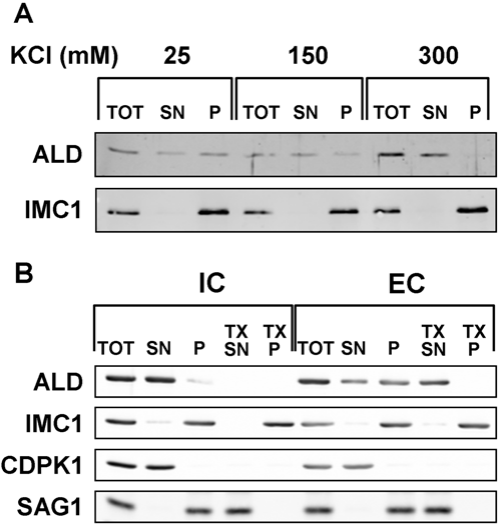
Pellicle-association of aldolase-1 in extracellular *Toxoplasma* tachyzoites. (A) Extracellular parasites were disrupted at 4°C by sonication in 25 mM MOPS pH 7.0 containing 5 mM MgCl_2_ and 25 mM, 150 mM, or 300 mM KCl and subsequently fractionated into soluble and insoluble fraction by centrifugation. Equal parasite equivalents of the starting material, supernatant, and pellet fractions were separated by SDS-PAGE and analyzed by immunoblotting with monospecific antisera to *Toxoplasma* aldolase-1 and the membrane skeleton protein IMC1. (B) Extracellular parasites and intracellular parasites were harvested, homogenized in 25 mM MOPS pH 7.0, 5 mM MgCl_2_ and 25 mM KCl as described above. The homogenate was fractionated into soluble and particulate material by centrifugation as above. The particulate fraction was subsequently extracted with 1% TX100 in the homogenization buffer and separated by centrifugation into detergent-soluble and insoluble fraction as described above. Equal parasite equivalents were analyzed by SDS-PAGe and immunoblotting with antisera to *Toxoplasma* aldolase-1, the membrane skeleton protein IMC1, the cytoplasmic protein CDPK1 and the plasma membrane protein SAG1. The starting material (TOT), supernatant (SN), and pellet (P) fractions were separated by SDS-PAGE and immuno-blotted with antisera to the indicated proteins.

Association of aldolase-1 with the pellicle in extracellular parasites does not appear to involve a covalent or otherwise stable interaction with the IMC membrane or associated cytoskeletal elements. When extracellular parasites are mechanically disrupted at approximate physiological salt concentrations (150 mM) and subjected to differential centrifugation, the majority of aldolase-1 is recovered in the supernatant. We noticed, however, that 20–30% of aldolase-1 was consistently recovered in the pellet fraction (Figure 9A). This association was sensitive to the salt concentration in the extraction buffer. When extractions were performed at higher KCl concentration (300 mM) all aldolase was recovered in the soluble fraction. At 25 mM KCl, on the other hand, we recovered 70–80% of aldolase-1 in the pellet fraction. To analyze this phenomenon in greater detail we compared the fractionation of aldolase-1 in intracellular and extracellular *Toxoplasma* at 25 mM KCl. As can be seen in Figure 9B, pellicle association of aldolase-1 is only observed in extracellular parasites under these conditions. The association is specific in that the cytoplasmic enzyme CDPK1 is completely solubilized under these conditions. Pellicle-association of aldolase-1 appears to require intact membranes as addition of Triton –X100 result in the complete solubilization of aldolase-1.

**Figure 10:**
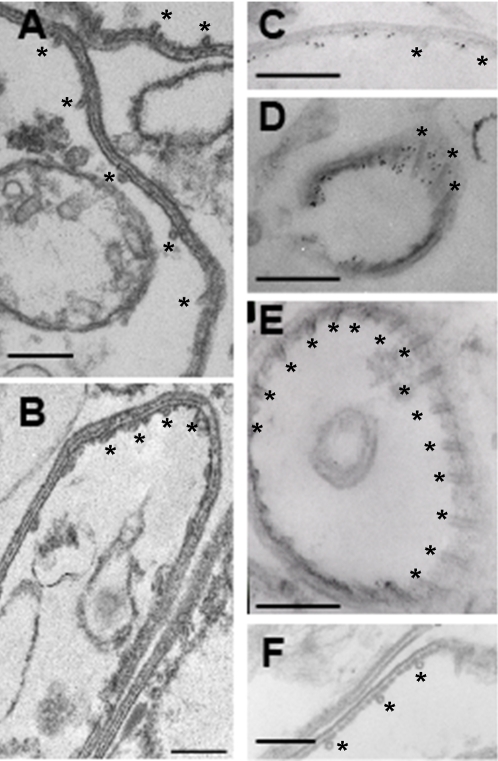
Aldolase-1 is associated with the cytoplasmic face of the IMC in extracellular *Toxoplasma*. *Toxoplasma* aldolase-1 is associated with the cytoplasmic face of the IMC. Intracellular (A, B) and extracellular (C–F) *Toxoplasma* tachyzoites were homogenized in 25 mM MOPS pH 7.0, 5 mM MgCl_2_ containing either 25 mM (A, C, D, F) or 300 mM KCl (B, E) and fractionated as described in Fig. 8A. Isolated pellicle fractions were incubated with antiserum to *Toxoplasma* aldolase-1 (A–E) or non-specific antiserum (F) and gold-conjugated secondary antibodies in the same extraction buffer and subsequently processed as described in Materials and Methods. Bars = 200 nm. Asterisks indicate the subpellicular microtubules on the cytoplasmic face of the IMC.

To determine if the salt-resistant fraction of aldolase-1 in extracellular *Toxoplasma* is indeed IMC associated, we homogenized parasites in 25 mM or 300 mM KCl buffer and performed pre-embedding immuno-electron microscopy on the isolated membrane fractions. It should be noted that association of the plasma membrane with the outer face of the IMC did not appear to be affected at the KCl concentrations used in this study. As can be seen in Figure 10 (panels A, B), no aldolase was detected in association with the pellicle of intracellular *Toxoplasma* prepared in 25 mM KCl. In pellicles isolated from extracellular parasites at 25 mM KCl labeling of the cytoplasmic face of the IMC was readily detected (Figure 10, panels C and D). This labeling was absent from pellicles in samples prepared in the presence of 300 mM KCl (Figure 10, panel E). Taken together with the data shown in Figures 3, 4, and 5 these findings indicate that a substantial fraction of aldolase-1 in extracellular *Toxoplasma* is intimately associated with the cytoplasmic face of the IMC membrane.

## Discussion

Obligate intracellular parasites like *Toxoplasma gondii* and other apicomplexa face vastly different environments during their normal life cycles in that they move between different host animals, different organ systems, and between the extracellular and intracellular milieu. It is therefore critical that these parasites have the ability to sense and adapt to changes in their environment. One of the most striking of these differences is found between extracellular and intracellular apicomplexan parasites. Whereas extracellular parasites are highly motile they are incapable of replication. Intracellular parasites, on the other hand, are non-motile but replicate efficiently with doubling times of as little as 8 hours. This observation suggests that *Toxoplasma* needs to have the ability to adjust numerous cellular processes that support these processes depending on the environment it finds itself in.

Considering its central role in all cellular processes, we reasoned that the production of ATP in *Toxoplasma* might be one process that would be tightly regulated with respect to overall activity and location as the parasite moves between the intracellular and extracellular environment. The *Toxoplasma* genome encodes all the enzymes required for energy production by glycolysis. It is not certain, however, if the parasite can generate energy by oxidative phosphorylation since the only recognizable homolog of pyruvate dehydrogenase appears to be targeted to the parasite's apicoplast where it is hypothesized to play a role in fatty acid biosynthesis [8],[9]. To determine the contribution of glycolysis and oxidative phosphorylation to the production of energy needed for *Toxoplasma* motility, we analyzed parasite movement under conditions where either glycolysis or the mitochondrial electron transport chain was blocked. It was clear from these experiments that glycolysis is responsible for supplying the majority of energy required for parasite movement (Figure 1).

Further analysis revealed that all glycolytic enzymes tested underwent a striking change in their cellular distribution after *Toxoplasma* egress from host cells (Figures 3, 8, and S5). Specifically, the enzymes were found throughout the parasite cytoplasm in intracellular *Toxoplasma* but relocated to the parasite pellicle after egress from the host cell. They remained at the parasite pellicle as long as parasites were extracellular or undergoing host cell invasion (Figure 5). In almost all cases, the glycolytic enzymes were distributed evenly along the pellicle of extracellular *Toxoplasma* and we did not detect any translocation of aldolase-1 along the anterior-to-posterior axis of the parasite during motility or host cell invasion (Figure 5A and 7D). The glycolytic enzymes reverted to a smooth cytoplasmic distribution upon completion of host cell invasion (Figure 6). While a fairly uniform cytoplasm-to-pellicle translocation was observed during *Toxoplasma* egress, the pellicle-to-cytoplasm translocation occurred over a variable period of time, ranging from several minutes up to several hours, but was essentially complete 4 hours after invasion. The observation that aldolase-1 distribution along the anterior-to-posterior axis is not obviously altered in motile or invading parasites differs from what was reported by Jewett and Sibley [1]. The reason for this difference is unclear at this time but it may well be that different detection reagents recognize different pools of aldolase-1.

Translocation of the glycolytic enzymes between the *Toxoplasma* cytoplasm and its pellicle is controlled by [K^+^] in the parasites' environment (Figure 7). Incubation of isolated parasites in a medium with a [K^+^] consistent with the intracellular milieu of animal host cells (∼145 mM) results in a cytoplasmic location of the enzymes tested. Transfer of these parasites to medium with a [K^+^] that is consistent with the extracellular milieu (∼5 mM) results in the rapid translocation of the enzymes to the parasite pellicle (Figure 7A). This translocation is completely reversed upon re-incubation of the parasites in media with intracellular levels of [K^+^]. We previously described that *Toxoplasma* egress is also activated in response to a transient increase in [Ca^2+^]_c_ that is triggered by a reduction of the [K^+^] in the decaying host cell [20]. The translocation of glycolytic enzymes from the parasite cytoplasm to its pellicle that occurs in response to lower [K^+^] also appears to require an increase in [Ca^2+^]_c_ as it is blocked by pre-incubation of parasites with BAPTA/AM (Figure 7C). The reverse process, in which the glycolytic enzymes are translocated from the *Toxoplasma* pellicle to its cytoplasm, is induced by the increase in [K^+^] experienced by the invading parasite, but this process does not require an increase in [Ca^2+^]_c_ in the parasite (Figure 7C).

The mechanism that brings about the actual translocation of the glycolytic enzymes between the *Toxoplasma* cytoplasm and its pellicle is not known at this time. Although Jewett and Sibley have demonstrated an interaction between aldolase-1 and F-actin, disruption of the latter with cytochalasin-D did not affect aldolase-1 translocation from the cytoplasm to the pellicle (Figure 7D). The translocation of glycolytic enzymes is also not affected by the disruption of parasite microtubules with oryzalin (Figure 7E). Although these observations rule out a major contribution of F-actin and microtubules in translocation of glycolytic enzymes in *Toxoplasma*, the possibility remains that a different active transport mechanism drives this process. Our observations are also consistent with a model that does not require such mechanisms, however. In this model, glycolytic enzymes diffuse freely through the cytoplasm of intracellular parasites. *Toxoplasma* egress and the accompanying increase in [Ca^2+^]_c_ would then result in the generation of binding sites for the glycolytic enzymes on the IMC, resulting in their eventual immobilization on that structure.

The specific mechanism through which the glycolytic enzymes are associated with the *Toxoplasma* pellicle is not clear at this time. The observation that association is disrupted at higher KCl concentrations suggests that it is not mediated by covalent modification of the protein by, for example, palmitoylation. The ease with which aldolase-1 is solubilized with detergents also argues against a high affinity interaction with the parasite's cytoskeleton. Instead, pellicle association appears to depend on ionic interactions with either lipids or proteins in the IMC membrane. It is interesting to note in this context that placement of a YFP tag at either the carboxy or amino-terminus of aldolase-1 disrupts relocation of the enzyme (data not shown). Although this effect could well be due to disruption of the tertiary or quaternary structure of the enzyme, it is also possible that the presence of a large fusion partner disrupts the interaction of aldolase-1 with factors that are critical for its translocation or pellicle association.

The pellicle of apicomplexan parasites consists of three membranes: the plasma membrane and the two membranes of the IMC. The space between the plasma membrane and IMC is only 18±0.6 nm wide (Figure S1) yet it houses multiple processes that are critical to parasite survival such as the myosin XIV motor complex [5], F-actin filaments [1], transporters for nutrients and ion homeostasis, as well as systems that control these processes. Sustained *Toxoplasma* motility and general survival therefore requires a reliable supply of ATP into the space between the parasite plasma membrane and IMC. As we have demonstrated here, the glycolytic apparatus of *Toxoplasma* tachyzoites is closely associated with the pellicle in extracellular parasites. Specifically, we have found that a substantial fraction of aldolase-1 is closely associated with the cytoplasmic face of the IMC. It is very important to note here that our data are not inconsistent with the presence of aldolase-1 in the space between the plasma membrane and IMC as proposed by Jewett and Sibley [1]. Given that these two structures remain intimately associated with each other during our subcellular fractionation experiments (Figure 10) it is unlikely that any aldolase-1 found in this space would be completely removed during extractions at higher KCl concentrations. This suggests then that only a small fraction of total aldolase-1 is likely to be present between the plasma membrane and IMC. This fraction could then be dedicated specifically to establishing an interaction between MIC2 and F-actin as proposed by Jewett and Sibley [1].

Our demonstration that *Toxoplasma* egress triggers a redistribution of its glycolytic enzymes from the cytoplasm to the IMC brings to mind the recent observation by Campanella et al [25] that multiple glycolytic enzymes, including aldolase, are intimately associated with the inner face of the plasma membrane of freshly isolated erythrocytes, but are cytoplasmic in deoxygenated cells [25]. These authors argued that close association of glycolytic enzymes with the erythrocyte plasma membrane could result in the generation of a highly compartmentalized pool of ATP, thus allowing for its direct and efficient consumption by e.g. nutrient or ion transporters. They also proposed that placing glycolytic enzymes in close proximity to each other at a membrane could improve the overall efficiency of ATP production by channeling reaction intermediates between individual enzymes. Our finding that glycolytic enzymes are associated with the pellicles of extracellular *Toxoplasma* tachyzoites indicates that ATP production in these organisms is also highly localized. In the case of *Toxoplasma* this would not only markedly improve ATP delivery to active transporter mechanisms in the parasite plasma membrane but also to its motile apparatus in the space between the plasma membrane and IMC. As was suggested for glycolysis in erythrocytes, the close association of glycolytic enzymes with the *Toxoplasma* pellicle could also markedly improve the overall efficiency of local ATP production by limiting the free diffusion of glycolytic reaction intermediates. This impact would be even greater if the glycolytic enzymes would physically associate with each other and thus allow for true substrate channeling. Although we have not been able to identify such complexes thus far, we do not rule out their existence, given that these interactions may well require labile cofactors or depend on specific but low-affinity interactions. Further analysis of the various enzymes is likely to shed light on this issue. Our experiments also need to be repeated on other members of the phylum Apicomplexa to determine if they extend to *Cryptosporidium* and *Plasmodium* species, causative agents of cryptosporidiosis and malaria.

## Materials and Methods

### *Toxoplasma* culture

*Toxoplasma gondii* tachyzoites of the RH(HX^−^) strain were grown in confluent monolayers of human foreskin fibroblasts (HFF) maintained in α minimal essential medium (αMEM; Invitrogen, Carlsbad, CA) supplemented with 2% fetal bovine serum (Hyclone, Logan, UT), 2mM glutamine, 100 IU/ml penicillin and 100 µg/ml streptomycin at 37°C with 5% CO_2_.

### Buffers

EC buffer: 120 mM NaCl, 1mM CaCl_2_, 5 mM MgCl_2_, 25 mM Hepes-NaOH pH 7.2, 1 mg/ml BSA. IC buffer: 142 mM KCl, 5 mM NaCl, 2 mM EGTA, 5 mM MgCl_2_, 25 mM Hepes-KOH pH 7.2, 1 mg/ml BSA. Where indicated, these buffers were supplemented 25 mM glucose, 25 mM 2-deoxyglucose, 2 mM KCN, or with 0.5 mM sodium pyruvate, 3 mM sodium lactate, 10 mM glutamine, or combinations thereof. IC-SUC buffer: 44.7 mM K_2_SO_4_, 10 mM MgSO_4_, 106 mM sucrose, 5 mM glucose, 20 mM Tris-H_2_SO_4_ pH 8.2, 1 mg/ml BSA.

### Production of antisera to *Toxoplasma* aldolase

The *Toxoplasma* aldolase-1 open reading frame was generated by PCR and cloned in pCR 2.1 TOPO (Invitrogen). The insert was recovered after digestion of the plasmid with BglII and EcoRI and ligated between the BglII and EcoRI sites of pRSET B (Invitrogen). Recombinant his_6_-tagged aldolase-1 was expressed in Escherichia coli BL21(DE3) strain (Stratagene, La Jolla, CA) and purified on a HisTrap Chelating HP column (Amersham Biosciences, Piscataway, NJ). The recombinant protein was used to immunize a mouse by subcutaneous injection (Cocalico Biologicals, Reamstown, PA). Anti-aldolase-1 antibodies were further purified by affinity chromatography on immobilized recombinant *Toxoplasma* aldolase.

### Expression constructs and transfection of *Toxoplasma*

The open reading frames of TgGT1, hexokinase (HK), aldolase-1 (ALD1), glyceraldehyde-3-phosphate dehydrogenase-1 (GAPDH1), pyruvate kinase-1 (PK1), and the tachyzoite lactate dehydrogenase-1 (LDH1) were amplified from *Toxoplasma gondii* cDNA (Thermoscript RTPCR, Invitrogen) using the following primer pairs: TgGT1, 5′-gacggatccatcATGGCGACGGAGGAGATGCG-3′ and 5′-atgcctaggAATACCACCTCCGTCCCCTTGG-3′; HK, 5′-cttagatctaaaATGCAGCCTCGTCAACCAGGC-3′ and 5′-tggcctaggcacTCAGTTCACATCTGCGATCAG-3′; ALD1, 5′-acaagatctaccATGTCGGGATACGGTCTTCCC-3′ and 5′-ctccctaggcgtTTAGTACACGTAGCGTTTCTC-3′; GAPDH1, 5′-cacagatctaagATGGTGTGCAAGCTGGG-3′ and 5′-taacctaggagcTTACGCGCCGTCCTGGACG-3′; PK1, 5′-ctcagatctacaATGGCATCTAAACAACCGC-3′ and 5′-ctgcctaggagtTTACTCCACAGTAAGAACC-3′; LDH1, 5′-tagagatctaaaATGGCACCCGCACTTGTGCAG-3′ and 5′-tttcctaggcgcTTACGCCTGAAGAGCAGCAAC-3′. The Bgl II, Bam HI, and Avr II restriction sites are underlined and non-coding sequences are shown in lowercase.

Isolated PCR products for HK, ALD1, GAPDH1, PK1, and LDH1 were digested with BglII and AvrII or BamHI and AvrII (TgGT1) and inserted between the BglII and AvrII sites of the vector pTG70, thus creating in-frame fusion between an N-terminal c-myc epitope and the different glycolytic enzymes. The PCR product for TgGT1 was digested with Bam HI and AvrII and ligated between Bgl II and Avr II sites of pTG168 thus creating an in-frame fusion between TgGT1 and a C-terminal myc epitope. Expression of the fusion proteins in both plasmids was driven by the α-tubulin promoter. After confirmation of all constructs by sequencing, they were transfected into *Toxoplasma* tachyzoites [5]. Stable transfectants expressing the myc-tagged protein of interest were selected by chloramphenicol as previously described [26].

### Aldolase-1 location after *Toxoplasma* egress

Infected HFF cells (24 h) were washed 3 times and mechanically disrupted on ice in ice-cold EC or IC buffer containing 25 mM glucose. Parasites were processed for immunofluorescence immediately after host cell egress (T_0_), or after an incubation of 15 minutes (T_15_) or 60 minutes (T_60_) at 37°C. Parasites that were incubated 60 minutes in IC buffer with glucose at 37°C were further washed 3 times in EC buffer with glucose, incubated 15 minutes (T_15_) or 60 minutes (T_60_) at 37°C and processed for immunofluorescence as described below.

### Aldolase-1 location during *Toxoplasma* invasion

Parasites expressing myc-aldolase-1 (5×10^6^ per coverslip) were allowed to invade host cell monolayers in complete medium for 2 minutes and subsequently incubated for 30 minutes, 2 hours, 4 hours, or 24 hours at 37°C. After two washes in PBS, cells were incubated for 30 minutes at 4°C with rabbit anti SAG1 antiserum BSA/PBS. After 3 washes in PBS, cells were fixed for 15 minutes with methanol at −20°C and processed for immunofluorescence as described below.

### Motility assays

The fraction of motile parasites, as well as the extent and consistency of parasite movement was optimal if parasites were released from infected host cells 30–32 hours after infection. At this stage, the vast majority (>95%) of parasites were intracellular. Infected HFF monolayers were typically scraped into IC buffer at 4°C. Parasites were released from cells by serial passage (3–4×) through a 18G needle and (2×) through a 25G needle. Parasites were recovered by centrifugation for 10 minutes at 600× g and resuspended in the buffer of choice. Parasites (5×10^6^) were placed on acid washed coverslips and incubated for 10 minutes at 25°C to allow for parasites attachment. Motility assays were performed for 15 minutes at 37°C. In experiments utilizing a nitrogen atmosphere, both the preincubation at 25°C and the actual motility assay at 37°C were performed in sealed vials flushed with dry nitrogen gas. Parasite motility was assessed by monitoring the trails of the cell surface antigen SAG1 deposited by moving parasites [27]. After fixation for 15 minutes in methanol at −20°C the parasites and deposited trails were visualized using a rabbit anti-SAG1 and Alexa_594_-conjugated goat-anti-rabbit IgG secondary antibodies. Length of individual trails was determined using ImageJ software (National Institutes of Health, Bethesda, MD). Parasites that had left a trail longer than 2 µm were considered motile, parasites with shorter or no trails were considered non-motile. In the vast majority of cases, trails left by motile parasites exceeded 10 microns.

### BAPTA/AM treatment

Host cells that were infected for 24 hours were washed 3 times in either IC buffer or IC-SUC buffer containing 25 mM glucose and either DMSO or 20 µM BAPTA/AM. Infected host cells were harvested in the same buffers and passed through a 25G needle to release the parasites from the parasitophorous vacuole. After a 30 minute incubation at 37°C, parasites were washed 3 times in EC buffer containing 25 mM glucose and either DMSO or 20 µM BAPTA/AM, incubated for 30 minutes at 37°C and processed for immunofluorescence.

Likewise, extracellular parasites were washed 3 times in EC buffer containing 25 mM glucose and DMSO or 20 µM BAPTA/AM, and incubated 30 minutes at 37°C. After 3 washes in IC or IC-SUC buffers containing 25 mM glucose and either DMSO or 20 µM BAPTA-AM, parasites were incubated 30 minutes at 37°C and processed for immunofluorescence.

### Cytochalasin D and oryzalin treatments

Cytochalasin D treatment was performed by incubating intracellular parasites for 15 minutes at 37°C with 10 µM drug. Infected host cells were disrupted by passing them through a 25G needle. Parasites were washed twice in EC buffer containing 25 mM glucose and 10 µM cytochalasin D before performing motility assay as described above but in the presence of 10 µM cytochalasin D. Oryzalin treatment was performed in the same manner except that drug was used at a concentration of 2.5 µM and intracellular parasites were treated for 24 hours.

### Cell fractionation

Parasites pellets (1×10^8^) were resuspended in 25 mM MOPS-KOH pH 7.0 containing 5 mM MgCl_2_ , protease inhibitors (Sigma), and 25 mM, 150 mM, or 300 mM KCl and disrupted by sonication for 2×20 seconds at 4°C using 50% power output and a continuous duty cycle (Branson Sonifier 450, Danbury, CT). Homogenates were centrifuged for 10 minutes at 14.000× g and 4°C. The pellets were resuspended in the same buffers containing 1% Triton X-100, incubated on ice for 5 minutes, and subjected to centrifugation as above. Samples of the original homogenate, the supernatant, and pellet fractions equivalent to 5×10^6^ parasites were separated by SDS-PAGE on 10% polyacrylamide gels, transferred to nitrocellulose, and probed with the indicated antibodies. Bound antibodies were visualized using a Li-Cor Odyssey Infrared Imaging System (Lincoln NE) and the appropriate secondary antibodies.

### Immunofluorescence microscopy

Extracellular *Toxoplasma* tachyzoites were allowed to adhere to acid-washed coverslips for 10 minutes at 4°C prior to fixation. Routinely, intracellular and extracellular parasites were fixed in cold methanol (−20°C) for 15 minutes at room temperature. Where indicated, parasites and parasite-infected cells were fixed instead in 3% formaldehyde and 0.01% glutaraldehyde in PBS for 15 minutes at 4°C. Coverslips were washed twice in PBS and remaining reactive aldehyde groups were neutralized with 1 mM NaBH_4_ in PBS for 10 minutes. After three washes in PBS cells were permeabilized for 10 minutes at 4°C with 1% TX100 in PBS. After 3 washes in PBS, samples were blocked with 3% BSA in PBS (BSA/PBS) for 15 minutes and subsequently incubated for 30 minutes with the indicated primary antibodies in BSA/PBS. After three washes in PBS, coverslips were incubated for 30 minutes with goat-anti-rabbit or goat-anti-mouse IgG conjugated to Alexa 488 or Alexa 594 (Molecular probes, Eugene, OR) and DAPI (5 ng/ml) in BSA/PBS. Coverslips were washed three times in PBS and mounted in MOWIOL. Cells were examined using a Nikon Eclipse TE2000-U epifluorescence microscope. Stacks of images were acquired using a Hamamatsu digital CCD camera (IEEE1394) directed by Metamorph (version 6.2r5, Sunnyvale, CA) software and processed for optical deconvolution by Auto deblur+Auto visualize program (version 9.3.3, Watervliet, NY) using 10 total iterations and low noise level for the algorithm settings.

### Immunoelectron microscopy

Extracellular and intracellular parasites (10^8^) were resuspended on ice in 25 mM MOPS-KOH pH 7.0 containing 5 mM MgCl_2_ , protease inhibitors (Sigma), and 25 mM or 300 mM KCl and disrupted by sonication as described above. The pellicle fraction was recovered by centrifugation for 10 minutes at 14.000× g and 4°C, resuspended in the same buffer and centrifuged again. The pellet fractions were resuspended in 3%BSA in the homogenization buffer containing mouse-anti-aldolase-1 or a nonspecific antiserum at 1∶50 dilution for 1 hour on ice followed by incubation with 10 nm gold-conjugated donkey anti-mouse IgG secondary antibody (Jackson Immuno Research Laboratories Inc., West Grove, PA) diluted 1/15 in the same buffer. Samples were washed 3 times in homogenization buffer and fixed in 1% glutaraldehyde in the same buffer for 1 hour at 4°C. After one wash in PBS, samples were incubated in 1% tannic acid in PBS for 20 minutes at 4°C. After 3 washes in 0.1 M sodium cacodylate pH 7.3, samples were treated with 1% osmium tetroxide in 0.1 M sodium cacodylate pH 7.3 for 20 min. After 3 washes in water, samples were dehydrated in increasing concentrations of ethanol and after 2 washes in propylene oxide, embedded in Epon. The polymerized blocks were sectioned at 60 nm with a Leica Ultracut UCT ultramicrotome (Leica Microsystems). Sections were stained with 2% uranyl acetate and Sato's lead stain and were viewed on an FEI Tecnai 12 electron microscope (FEI, Hillsboro, OR). Images were collected with a Gatan model 794 multiscan digital camera (Gatan, Pleasanton, CA).

## Supporting Information

Figure S1 Distances between membranes of the *Toxoplasma* pellicle. Extracellular *Toxoplasma* tachyzoites were harvested and processed for electron microscopy as described. Sections were analyzed at 70,000× magnification and 4 images of pellicle of each of 4 parasites from two separate preparations of parasites were collected. Distances between the plasma membrane and the outer and inner membranes of the inner membrane complex were determined by plotting the optical density along lines drawn perpendicular to the pellicle membranes and determining the distances between the centers of the lipid bilayers. Panel A shows a representative image used for this analysis. Two parasites are shown lying side-by-side. Panel B shows a diagram of the *Toxoplasma* pellicle structure. Panel C shows the optical density profile of a line scan performed on the image in panel A. Panel D shows the results of the analysis (±S.D.).
(1.40 MB TIF)

Figure S2 Antisera reactivity with *Toxoplasma* aldolase-1. (A) Purified rabbit (Rb) aldolase and purified his6-tagged recombinant *Toxoplasma* (Tg) aldolase-1 were separated by SDS-PAGE and transferred to nitrocellulose membranes. The same membrane was first stained with Ponceau S to visualize the proteins (left panel) and subsequently incubated with commercial goat-anti-rabbit aldolase (Chemicon, right panel). The membrane incubated with goat-anti-rabbit aldolase was exposed overnight. Molecular weights are in kDa. (B) Purified rabbit aldolase (ALD; 100 ng), purified recombinant his_6_-tagged *Toxoplasma* aldolase-1 (100 ng), total protein extracts of RH parasites (2×10^6^ parasites) and a RH parasite strain expressing myc-tagged aldolase-1 (2×10^6^ parasites) were separated in parallel by SDS-PAGE and analyzed by immunoblot using either pre-immune serum (left membrane), anti-*Toxoplasma* aldolase-1 antiserum (middle membrane) or anti-myc monoclonal antibodies (right membrane). The slightly higher MW_app_ of the recombinant compared to endogenous *Toxoplasma* aldolase-1 is due to the presence of the his_6_ and Xpress™-tags on the former. Molecular weights are indicated in kDa.
(1.45 MB TIF)

Figure S3 Localization of aldolase-1 and MIC2 in motile parasites. Parasites expressing myc-tagged aldolase-1 were used for motility assays and after fixation in −20°C methanol analyzed by immunofluorescence microscopy using anti-myc (mALD1, green) and anti-MIC2 (red) antibodies. A DIC image is shown on the right. Bars = 2 µm.
(0.93 MB TIF)

Figure S4 Localization of aldolase-1 in intracellular parasites. Immunofluorescence of 2, 4, 8 or 32 parasites per parasitophorous vacuole with details of green, red, blue channels and DIC corresponding to overlay pictures shown in Figure 4C. Parasites expressing myc-tagged aldolase-1 were fixed in −20°C methanol and labeled with mouse anti myc (mALD, green), rabbit anti IMC1 (red) and DAPI (blue). Overlay pictures of DIC and DAPI are represented on the right. Bars = 2 µm.
(4.37 MB TIF)

Figure S5 Localization of hexokinase, GAPDH1, pyruvate kinase-1 and LDH1 in *Toxoplasma* tachyzoites. Intracellular and extracellular *Toxoplasma* tachyzoites expressing myc-tagged versions of hexokinase, GAPDH1, pyruvate kinase-1, and LDH1 were fixed in −20°C methanol and processed for immunofluorescence microscopy using antibodies to the myc-epitope (green) and IMC1 (red). DAPI (blue) was used to label parasite nuclei. Overlay pictures of DIC and DAPI images are shown on the right. Bars = 2 µm.
(6.97 MB TIF)
