# Supplementary figures and images for: Host Cell Egress and Invasion Induce Marked Relocations of Glycolytic Enzymes in Toxoplasma gondii Tachyzoites
Source: PLoS Pathog. 2008 Oct 24;4(10):e1000188. doi: 10.1371/journal.ppat.1000188 (PMC2563030; doi:10.1371/journal.ppat.1000188)

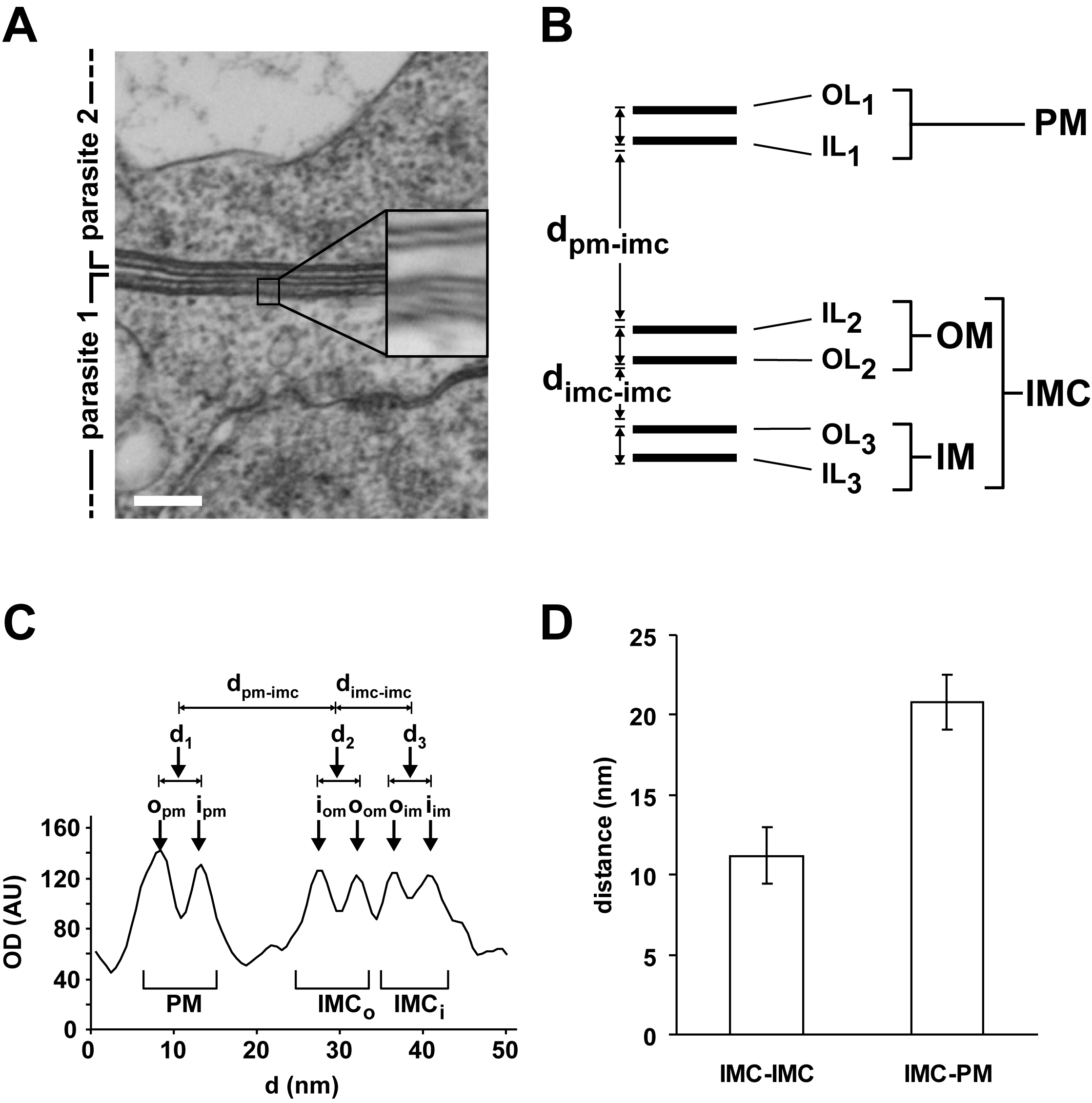

Supplement: Figure S1 — Distances between membranes of the Toxoplasma pellicle. Extracellular Toxoplasma tachyzoites were harvested and processed for electron microscopy as described. Sections were analyzed at 70,000× magnification and 4 images of pellicle of each of 4 parasites from two separate preparations of parasites were collected. Distances between the plasma membrane and the outer and inner membranes of the inner membrane complex were determined by plotting the optical density along lines drawn perpendicular to the pellicle membranes and determining the distances between the centers of the lipid bilayers. Panel A shows a representative image used for this analysis. Two parasites are shown lying side-by-side. Panel B shows a diagram of the Toxoplasma pellicle structure. Panel C shows the optical density profile of a line scan performed on the image in panel A. Panel D shows the results of the analysis (±S.D.). (1.40 MB TIF) [file ppat.1000188.s001.tif]

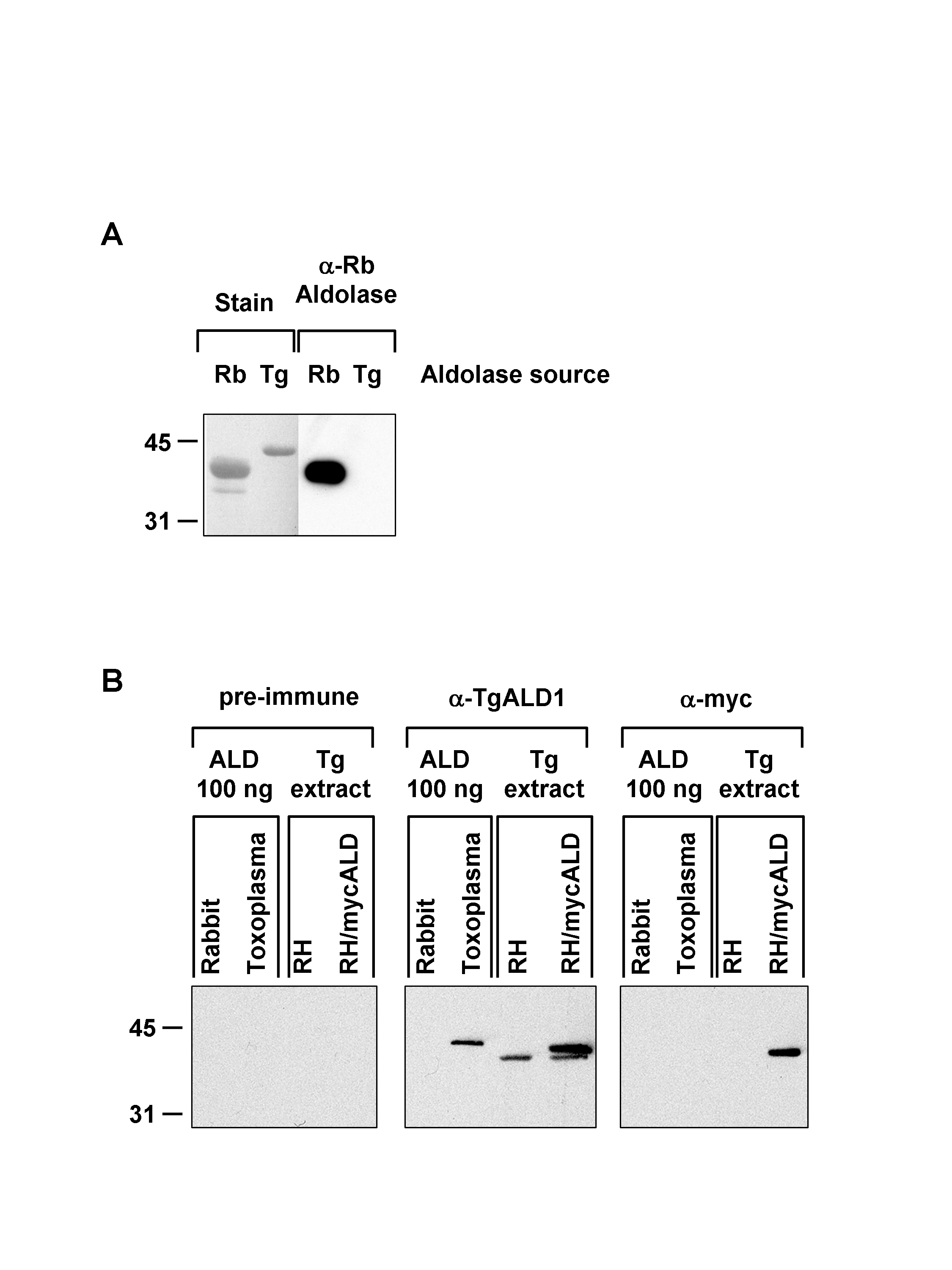

Supplement: Figure S2 — Antisera reactivity with Toxoplasma aldolase-1. (A) Purified rabbit (Rb) aldolase and purified his6-tagged recombinant Toxoplasma (Tg) aldolase-1 were separated by SDS-PAGE and transferred to nitrocellulose membranes. The same membrane was first stained with Ponceau S to visualize the proteins (left panel) and subsequently incubated with commercial goat-anti-rabbit aldolase (Chemicon, right panel). The membrane incubated with goat-anti-rabbit aldolase was exposed overnight. Molecular weights are in kDa. (B) Purified rabbit aldolase (ALD; 100 ng), purified recombinant his6-tagged Toxoplasma aldolase-1 (100 ng), total protein extracts of RH parasites (2×10^6^ parasites) and a RH parasite strain expressing myc-tagged aldolase-1 (2×10^6^ parasites) were separated in parallel by SDS-PAGE and analyzed by immunoblot using either pre-immune serum (left membrane), anti-Toxoplasma aldolase-1 antiserum (middle membrane) or anti-myc monoclonal antibodies (right membrane). The slightly higher MWapp of the recombinant compared to endogenous Toxoplasma aldolase-1 is due to the presence of the his6 and Xpress™-tags on the former. Molecular weights are indicated in kDa. (1.45 MB TIF) [file ppat.1000188.s002.tif]

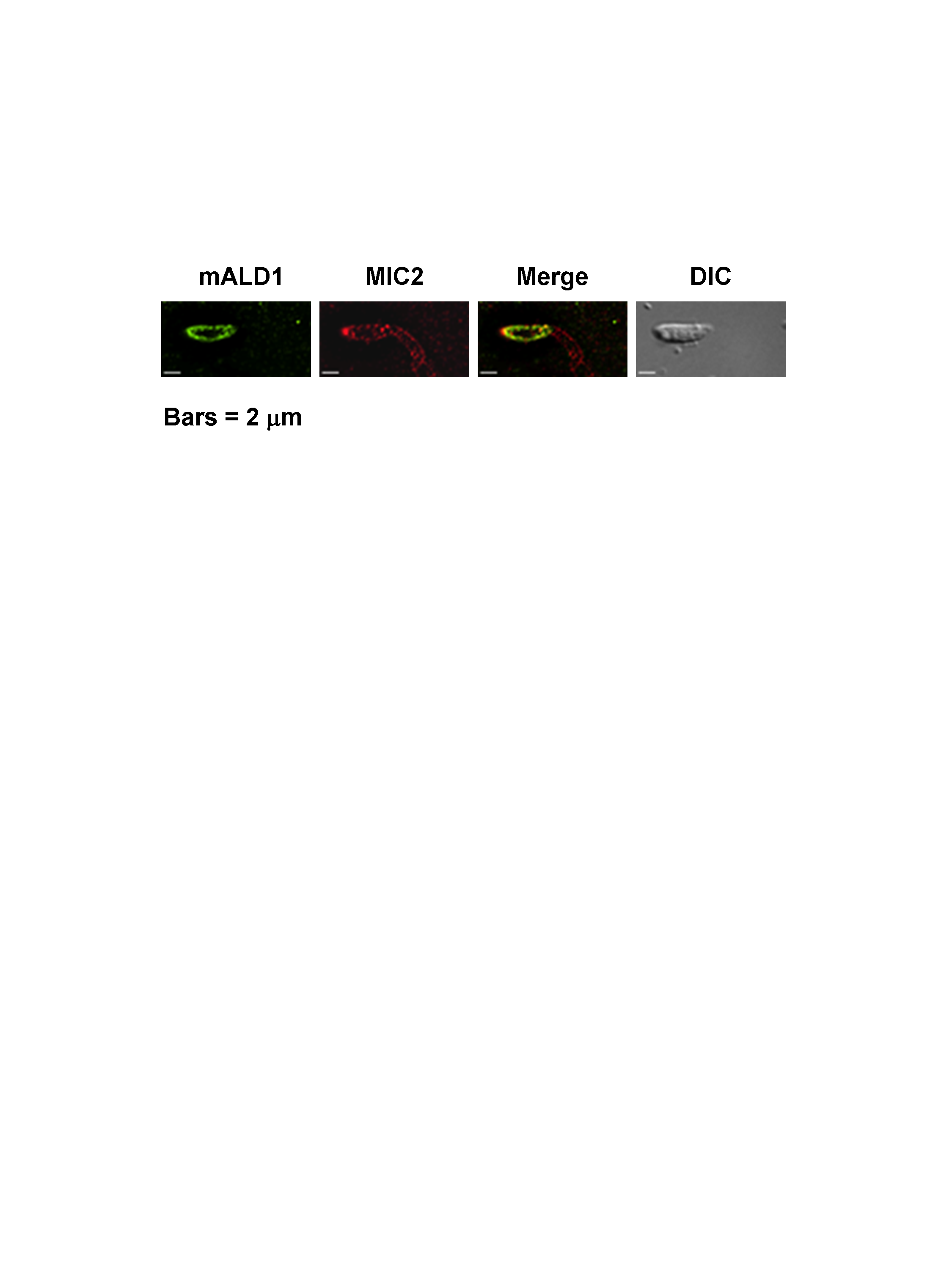

Supplement: Figure S3 — Localization of aldolase-1 and MIC2 in motile parasites. Parasites expressing myc-tagged aldolase-1 were used for motility assays and after fixation in −20°C methanol analyzed by immunofluorescence microscopy using anti-myc (mALD1, green) and anti-MIC2 (red) antibodies. A DIC image is shown on the right. Bars = 2 µm. (0.93 MB TIF) [file ppat.1000188.s003.tif]

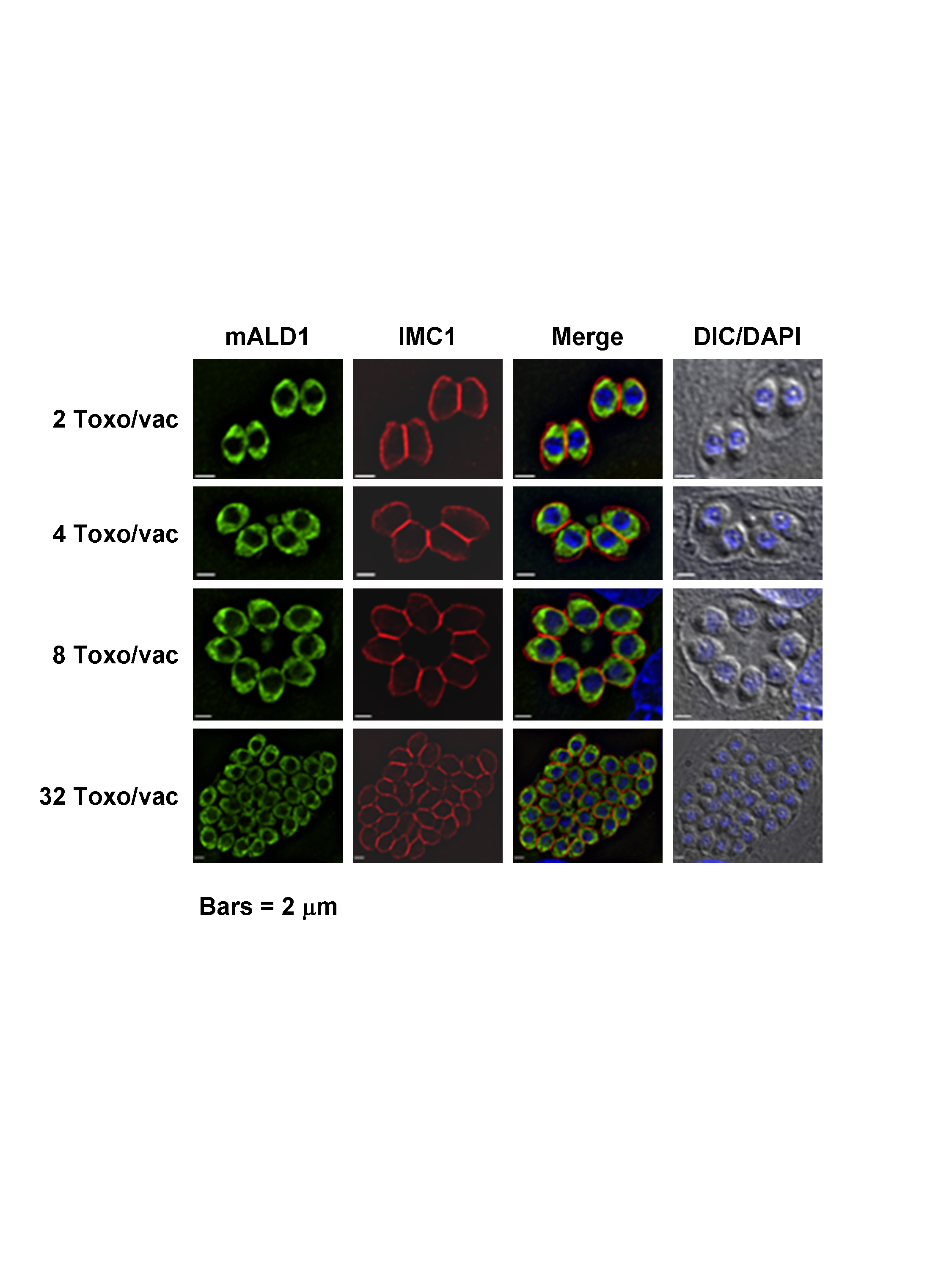

Supplement: Figure S4 — Localization of aldolase-1 in intracellular parasites. Immunofluorescence of 2, 4, 8 or 32 parasites per parasitophorous vacuole with details of green, red, blue channels and DIC corresponding to overlay pictures shown in Figure 4C. Parasites expressing myc-tagged aldolase-1 were fixed in −20°C methanol and labeled with mouse anti myc (mALD, green), rabbit anti IMC1 (red) and DAPI (blue). Overlay pictures of DIC and DAPI are represented on the right. Bars = 2 µm. (4.37 MB TIF) [file ppat.1000188.s004.tif]

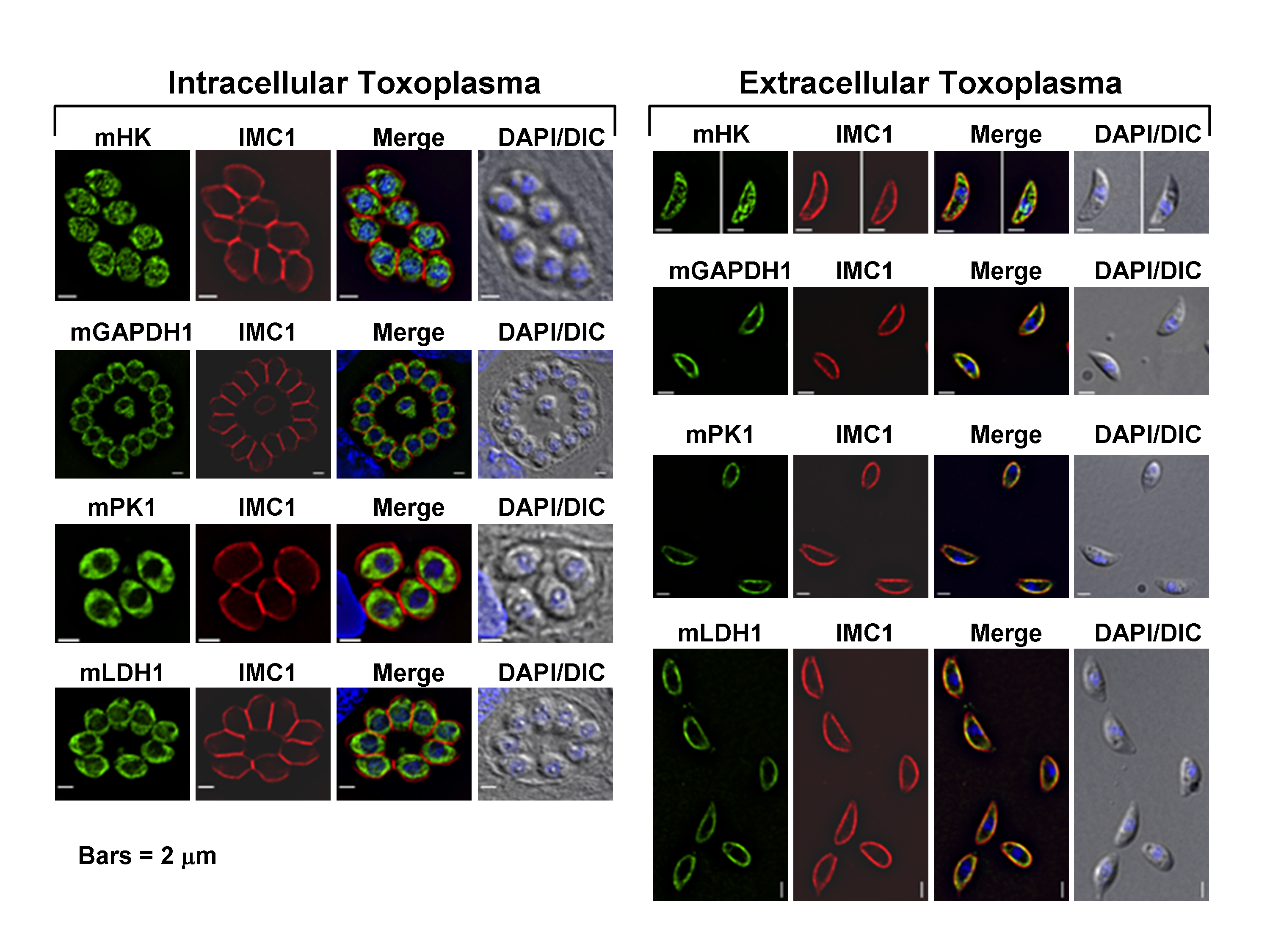

Supplement: Figure S5 — Localization of hexokinase, GAPDH1, pyruvate kinase-1 and LDH1 in Toxoplasma tachyzoites. Intracellular and extracellular Toxoplasma tachyzoites expressing myc-tagged versions of hexokinase, GAPDH1, pyruvate kinase-1, and LDH1 were fixed in −20°C methanol and processed for immunofluorescence microscopy using antibodies to the myc-epitope (green) and IMC1 (red). DAPI (blue) was used to label parasite nuclei. Overlay pictures of DIC and DAPI images are shown on the right. Bars = 2 µm. (6.97 MB TIF) [file ppat.1000188.s005.tif]
